# Validation of Suitable Reference Genes for Expression Studies in Different Pilocarpine-Induced Models of Mesial Temporal Lobe Epilepsy

**DOI:** 10.1371/journal.pone.0071892

**Published:** 2013-08-23

**Authors:** Thalita Ewellyn Batista Sales Marques, Leila Rodrigues de Mendonça, Marília Gabriela Pereira, Tiago Gomes de Andrade, Norberto Garcia-Cairasco, Maria Luisa Paçó-Larson, Daniel Leite Góes Gitaí

**Affiliations:** 1 Department of Cell, Molecular Biology, Institute of Biological Sciences and Health, Federal University of Alagoas, AL, Brazil; 2 Department of Physiology, Ribeirão Preto School of Medicine, University of São Paulo, Ribeirão Preto, São Paulo, Brazil; 3 Campus Arapiraca, Federal University of Alagoas, AL, Brazil; 4 Department of Cellular and Molecular Biology, Ribeirão Preto School of Medicine, University of São Paulo, Ribeirão Preto, São Paulo, Brazil; Aston University, United Kingdom

## Abstract

It is well recognized that the reference gene in a RT-qPCR should be properly validated to ensure that gene expression is unaffected by the experimental condition. We investigated eight potential reference genes in two different pilocarpine PILO-models of mesial temporal lobe epilepsy (MTLE) performing a stability expression analysis using geNorm, NormFinder and BestKepeer softwares. Then, as a validation strategy, we conducted a relative expression analysis of the *Gfap* gene. Our results indicate that in the systemic PILO-model *Actb, Gapdh, Rplp1, Tubb2a* and *Polr1a* mRNAs were highly stable in hippocampus of rats from all experimental and control groups, whereas *Gusb* revealed to be the most variable one. In fact, we observed that using *Gusb* for normalization, the relative mRNA levels of the *Gfap* gene differed from those obtained with stable genes. On the contrary, in the intrahippocampal PILO-model, all softwares included *Gusb* as a stable gene, whereas *B2m* was indicated as the worst candidate gene. The results obtained for the other reference genes were comparable to those observed for the systemic Pilo-model. The validation of these data by the analysis of the relative expression of *Gfap* showed that the upregulation of the *Gfap* gene in the hippocampus of rats sacrificed 24 hours after status epilepticus (*SE*) was undetected only when *B2m* was used as the normalizer. These findings emphasize that a gene that is stable in one pathology model may not be stable in a different experimental condition related to the same pathology and therefore, the choice of reference genes depends on study design.

## Introduction

Uncovering the molecular mechanisms involved in ictogenesis and epileptogenesis is critical to understand the physiopathology of epilepsies and for developing new therapeutic options. An approach that has been widely used is the analysis of differential gene expression in the affected tissue [1]–[4]. Quantitative real-time PCR (RT-qPCR) is currently the gold standard for the quantification of steady-state mRNA levels due to its accuracy and sensitivity [5]–[7]. However, in this type of analysis, an appropriate normalization strategy is required for the correction of experimental variations introduced by pipetting errors, inhibitory compounds, reverse transcription efficiency or quality of starting material [8]. At present, the most common method for such normalization is the use of endogenously expressed control genes - also known as “reference or housekeeping genes” [9].

Ideally, reference genes should present high expression stability levels in different experimental conditions [10], [11]. Genes related to basic and structural processes in the cell (beta-actin, glyceraldehyde-3-phosphate dehydrogenase, ribosomal subunits, beta-tubulin, and others) have been used directly as normalizers in quantitative assays. However, there are strong evidences in the literature suggesting that expression of these types of genes vary between cell types and experimental conditions [12]–[14]. The impact of using an unstable internal control can lead to inaccurate results and erroneous conclusions. It is essential, therefore, to identify and validate the reference gene prior to its use for normalization during specific experimental set ups.

Many of human Mesial Lobe Temporal Epilepsy (MTLE) characteristics can be reproduced in rodents by injection of pilocarpine (PILO). In this animal model, SE is followed by a latent period and later by the appearance of spontaneous recurrent seizures (SRSs) [15]–[18]. A large amount of expression gene data obtained at distinct time points corresponding to the latent to chronic phase transition of the PILO-model has been reported. Surprisingly, with one exception [19] as far as we know, reference genes are almost always used as internal controls without any preliminary evaluation of their suitability. In fact, combining different terms, such as “gene expression”, “pilocarpine”, ‘‘epilepsy” and “PCR”, we performed a PubMed search for articles published from January 1, 2005 to February 1, 2012 and got 43 available articles that evaluated gene expression changes by RT-PCR in the PILO-model. None of the studies used validated (or multiples) reference genes for data normalization. *Gapdh* was the normalizer gene most frequently used (56.1%), followed by *Actb* (22%), *Syp* (9.8%), *Ppia* (7.3%) and *Rn18S* (4.9%).

Only recently, candidate reference genes have been proposed for gene expression studies in a kainate model of MTLE, and in human epileptic brain tissue [20]–[22]. The sole study using the PILO-model restricted the assessment to the chronic phase [23].

The present study was thus designed to define further suitable reference genes for expression analysis in epileptogenesis induced in two different PILO-models of MTLE. The gene expression levels of eight commonly used housekeeping genes (beta-actin (*Actb*), beta-2-microglobulin (*B2m*), glyceraldehyde-3-phospate dehydrogenase (*Gapdh*), beta-glucuronidase (*Gusb*), beta-tubulin (*Tubb2a*), peptidylprolyl isomerase A (*Ppia*), ribosomal protein, large, P1 (*Rplp1*) polymerase (RNA) I, polypeptide A (*Polr1a*) were investigated in the hippocampus of experimental and control animals. The expression stability was analyzed, independently, with the geNorm [24], NormFinder [25], and BestKeeper [26] softwares. Finally, as a validation strategy, we used each one of the candidate reference genes to measure PILO-induced changes in glial fibrillary acidic protein (*Gfap*) mRNA, a gene whose expression pattern variation in PILO injected model is known.

## Materials and Methods

### Animals

Experiments were conducted on Wistar male rats (n = 49). From those: 37 (systemic PILO, n = 31; controls, n = 6) from the main breeding stock of the Federal University of Alagoas and 12 (intra-hippocampal PILO, n = 6; controls (n = 6) from the main breeding stock of the University of São Paulo, Campus of Ribeirão Preto. All rats were 90–100-days-old and weighted from 200 to 250 g. They were kept at 22°C in groups of four per cage with free access to food and water, in a 12-h light/dark cycle (lights on at 08:00 h). All experimental procedures were performed according to the Brazilian Society for Neuroscience and Behavior, which are based on international guidelines of the ethical use of animals, such as those from the Society for Neuroscience. The systemic PILO protocol was approved by the Research Ethics Committee of the Federal University of Alagoas (Permit number: 011462/2010-83), and the intra-hipocampal PILO work was approved by the Committee on the Ethics of Animal Experiments of the Ribeirão Preto Medical School of the University of São Paulo (Permit number: 195/2005). All efforts were made to minimize the number of animals used and to avoid any unnecessary suffering.

### Surgery

Animals were deeply anesthetized with 10 ml/kg of tribromo ethanol (2.5%; Aldrich Chemical Inc., Milwaukee, WI, USA), followed by veterinary pentabiotic, 1 ml/kg (Fort Dodge, Campinas, SP, Brazil) to avoid infection. Cannulae were implanted in specific stereotaxic coordinates [27] hilus of the DG: −6.30 mm anterior–posterior (AP, reference; bregma), 4.50 mm medial–lateral (ML, reference: sagittal sinus), −4.50 mm dorsal–ventral (DV, reference: dura mater).

### Intrahippocampal PILO microinjections

Animals were gently restrained during the intrahippocampal microinjection. A 5 μl syringe (Hamilton Company, Reno, NV, USA) connected to a microinjection pump (Harvard Apparatus PHD 2000, Holliston, MA, USA) was used. The total injected volume was 1 μl, dose of 2.4 mg/μl at a speed of 0.5 μl/min. The experimental group (n = 6) was injected with pilocarpine, and the control group (n = 6) was injected with saline (0.9%; 1 μl). The PILO injected animals had SE and were rescued with diazepam (DZP; 5 mg/kg; ip) 90 minutes after SE establishment. For this experimental group we have used 5 animals that yielded RNA of good quality. Control groups animals were also injected with DZP in the same conditions. Animals were sacrified twenty four hours after SE. Only the contra lateral hippocampus was used for gene expression analysis.

### Systemic PILO injections

Animals were injected intra-peritoneally with scopolamine butyl bromide (1 mg/kg) in order to reduce peripheral cholinergic effects, followed after 30 min by PILO in a dose of 320 mg/kg. All animals that had SE were rescued with DZP (5 mg/kg; ip), 90 min after SE establishment. Out of 31 PILO-injected rats, 14 died during the experiments, and 17 developed SE and survived. From the third day after SE, animals (chronic group) were individually placed in acrylic cages and their behavior was recorded on videotapes for up to 6 hours per day. Four groups of rats subjected to SE were used: animals sacrificed immediately (n = 6), twenty four hours (n = 6) and 10 weeks after SE (n = 5). All animals from this last group showed, two or more SRS with seizure severity scores equal or greater than 3, according to the scale of Racine [28] and Pinel and Rovner [29]. Naive rats were used as control group (n = 6).

### RNA extraction and cDNA synthesis

Rats were guillotined and the brains were immediately dissected on ice. Hippocampi were rapidly frozen and stored in liquid nitrogen until RNA isolation. Total RNA was purified using Trizol reagent (Invitrogen, CA, USA), following the manufacturers protocol. The quality of total RNA was assessed by analysis of the ratio of 28S to18S ribosomal RNAs after electrophoresis in 1% agarose gel. Total RNA was treated with DNase I (Ambion, TX, USA) for 30 min in order to avoid amplification of genomic DNA.

Total RNA (1 µg) was converted to first-stranded cDNA using High Capacity® Kit (Applied Biossystems, CA, USA), as recommended by the manufacturer. All samples were diluted (10X) in TE (Tris 10 mM, pH 7,4; EDTA 0,1 mM, pH 8,0) and stored at –80°C until further analysis.

### Quantitative cDNA amplification by real time PCR

Eight commonly used reference genes, *Actb, B2m, Gapdh, Gusb, Tubb2a, Ppia, Rplp1, Polr1a* and one target gene, *Gfap*, were used (Table 1).

**Table 1:** Primer sequences and amplification summary.

Gene*	Reference	5′–3′ sequence	Amplicon length (bp)	Final Concentration (μM)	PCR efficiency (%)
*Tubb2a*	NM_001109119.1	F – TTGTGTTCGGTCAGAGTGGT	103	0.4	95.46
		R – GACTCCTTCCTCACCACATC			
*B2m*	NM_012512.1	F– ATCTTTCTGGTGCTTGTCTCT	140	0.4	98.81
		R – TGAGGTGGGTGGAACTGAGA			
*Actb*	NM_031144.2	F – AGCCTTCCTTCCTGGGTATG	92	0.2	96.61
		R – GAGGTCTTTACGGATGTCAAC			
*Gapdh*	NM_017008.3	F– CCCATTCTTCCACCTTTGATGCT	104	0.4	96.57
		R– CTGTTGCTGTAGCCATATTCAT			
*Gusb*	NM_017015.2	F – CCGTGGAACAGGGAATGAG	121	0.4	99.70
		R – CTCAGGTGTTGTCATCGTCA			
*Ppia*	NM_017101.1	F– AGCACTGGGGAGAAAGGATT	174	0.6	100.07
		R– GATGCCAGGACCTGTATGCT			
*Polr1a*	NM_031772.1	F – CAGGAGAAGTGCCTGAGACC	188	0.4	92.47
		R – TCCTCCTCTCTCCGATTCCT			
*Rplp1*	NM_001007604.1	F – GCATCTACTCCGCCCTCA	58	0.2	95.79
		R – ATCTTATCCTCCGTGACCGT			
*Gfap*	NM_017009.2	F – AACCGCATCACCATTCCTGT	123	0.2	91.18
		R – CATCTCCACCGTCTTTACCAC			

* *Tubb2a*, tubulin beta 2A class IIa; *B2m*, β-2-Microglobulin; *Actb*, β-Actin; *Gapdh*, Glyceraldehyde-3-phospate dehydrogenase; *Gusb*, β-Glucuronidase; *Ppia*, peptidylprolyl isomerase A; *Porla1a*, polymerase (RNA) I polypeptide A; *Rplp1*, ribosomal protein, large, P1; *Gfap*, glial fibrillary acidic protein.

Real-time analysis was carried out on StepOnePlus™ Real Time PCR systems (Applied Biosystem, CA, and USA). Reactions were performed in a 12 μL volume containing cDNA (2,5 μL), 0.2–0,6 μM each of specific forward (F) and reverse (R) primers, and 6 µl Power Syber® Green PCR Master Mix (Applied Biosystem, CA, USA). Primers were designed to span an exon–intron boundary to exclude amplification of genomic DNA. Selected forward and reverse primer sequences and characteristics are listed in Table 1. The amplification protocol used was as follows: initial 10 min denaturation and 40 cycles of 95°C for 15s and 60°C for 1 min. These cycles were followed by a melting-curve analysis, ranging from 60°C to 95°C, with temperature increases in steps of 0.5°C every 10 s. The absence of contamination was confirmed by PCR amplification in the absence of cDNA. Each assay was performed in duplicate and the mean values were used for further analysis. To estimate the efficiencies of amplification, a standard curve was generated for each primer pair based on 5 points of serial dilution of pooled cDNA (1:20; 1:40; 1:80; 1:160 and 1:320). Mean threshold cycle (Tc) values of each two-fold dilution were plotted against the logarithm of the cDNA dilution factor. An estimate of PCR efficiency was derived from the expression [10(1/-S)–1]×100%, where S represents the slope of the linear regression [6]. All calibration curves exhibited correlation coefficients higher than 0.99 and the corresponding real-time PCR efficiencies were in the range 0.90–1.10 (Table 1).

### Determination of reference gene expression stability

To assess the stability of candidate reference genes, 3 commonly used approaches geNorm (http://medgen.ugent.br/~jvdesomp/genorm/), NormFinder (http://www.mdl.dk/publicationsnormfinder.html) and Bestkeeper (http://www.wzw.tum.de/gene-quantification/bestkeeper.html) algorithms were utilized.

In geNorm and NormFinder, Ct values were converted into relative quantities via the delta-Ct method using the sample with the lowest Ct as calibrator, accordling with the 2^–ΔCt^ method [30]. For Bestkeeper program, the raw Ct values were used.

GeNorm uses an algorithm to calculate the M value, a gene expression stability factor, defined as the mean pairwise variation for a given gene compared to the remaining tested genes. Hence, a lower M value indicates higher stability of the reference gene. We considered 0.5 as a cut-off for M value because it is the smallest value that is higher than the stability values of all reference genes tested [24]. The program also estimates the pairwise variation between two sequential calculations of normalization factors (NF) including an increasing number of genes. This defines the minimal number of genes required to calculate a robust normalization factor. NormFinder uses an ANOVA-based model to estimate intra- and inter-group variation, and combines these estimates to provide a direct measure of the variation in expression for each gene. Bestkeeper generates an index using the geometric mean of the Ct values of best candidate genes under study. This index was then compared to each individual candidate housekeeping gene by pair-wise correlation analyses, with each combination assigned a value for the Pearson correlation coefficient (r) and the probability (p).

### Reference gene validation

*Gfap* transcripts were used as target gene in order to validate the best reference genes for normalization of relative expression in epileptogenesis induced by PILO. Its relative quantity in each sample was normalized either to the most stable combination, in accordance with geNorm and NormFinder analyses, or to each of the eight reference genes independently, using the 2^ΔΔCt^ method [31].

For each normalization strategy, the *Gfap* relative expression was statistically compared among the different animal groups using nonparametric ANOVA or T-test, followed by appropriate post hoc analysis using GraphPad Prism version 5.00. A p value of less than 0.05 was accepted as significant.

## Results

### Transcription profile

**Figure 1:**
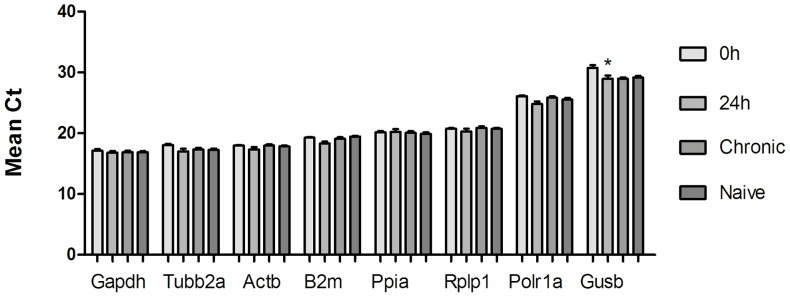
Expression levels of the candidate reference genes across experimental samples. Values are given in the form of RT-qPCR threshold cycle numbers (Ct values), mean ± SD (n = 6), * 0 h compared with 24 h, p<0.05.

Figure 1 gives the mean of Ct values for each gene in the hippocampus of systemic-PILO-injected and naive rats, illustrating the expression levels among the different experimental groups. The eight candidate reference genes displayed a relatively wide expression range, with mean Ct values between 16.96 (*Gapdh*) and 29.45 (*Gusb*). When the reference genes were grouped into two arbitrary categories using the mean Ct value at 21 cycles, the lower-expression group included *Gusb* and *Polr1a*. Using ANOVA, only *Gusb* was observed to be differently expressed between the 0h and 24 hour groups (P = 0.0286) of PILO-model (Figure 1).

### Stability expression analysis

In order to determine the expression stability of selected reference genes during different periods of the epileptogenic process, we used geNorm, Normfinder and Bestkeeper softwares, separately.

### GeNorm Analysis

**Figure 2:**
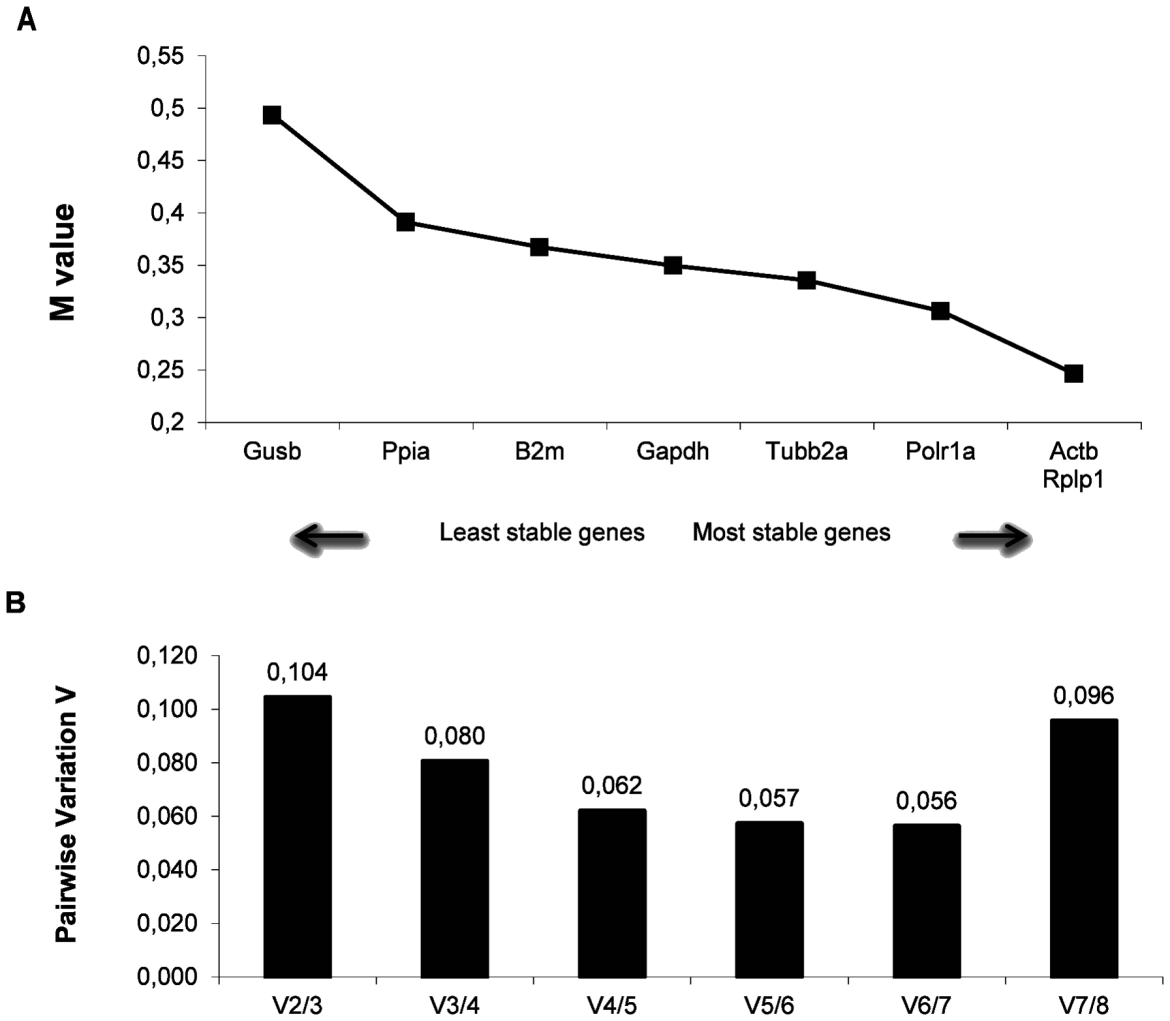
Selection of the most suitable reference genes for normalization in systemic PILO-model samples using geNorm analysis. **A)** Expression stability measures (M) of the eight reference genes analyzed. The x-axis from left to right indicates the ranking of the genes according to their expression stability; lower M values indicate higher expression stability. **B)** Determination of the optimal number of reference genes for normalization was conducted. The software calculates the normalization factor from at least two genes at which the variable V defines the pair-wise variation between two sequential normalization factors.

The average expression stability values (M values) of the eight reference genes in all tested samples from systemic PILO injected rats are displayed in Figure 2a. All the genes presented high expression stability, with the M values varying between 0.24 and 0.49. To determine the minimum number of reference genes necessary for an accurate normalization, a pairwise variation Vn/n+1 analysis was performed (Figure 2b). GeNorm defines a pairwise variation of 0.15 as the cutoff value, below which the inclusion of an additional reference gene is unnecessary [24]. Here, the V2/3 value was 0.104 which was below the cutoff value; thus the *Actb*/*Rplp1* genes were indicated as the optimal pair to provide normalization of gene expression in the different points of tested epileptogenesis.

### Normfinder Analysis

**Figure 3:**
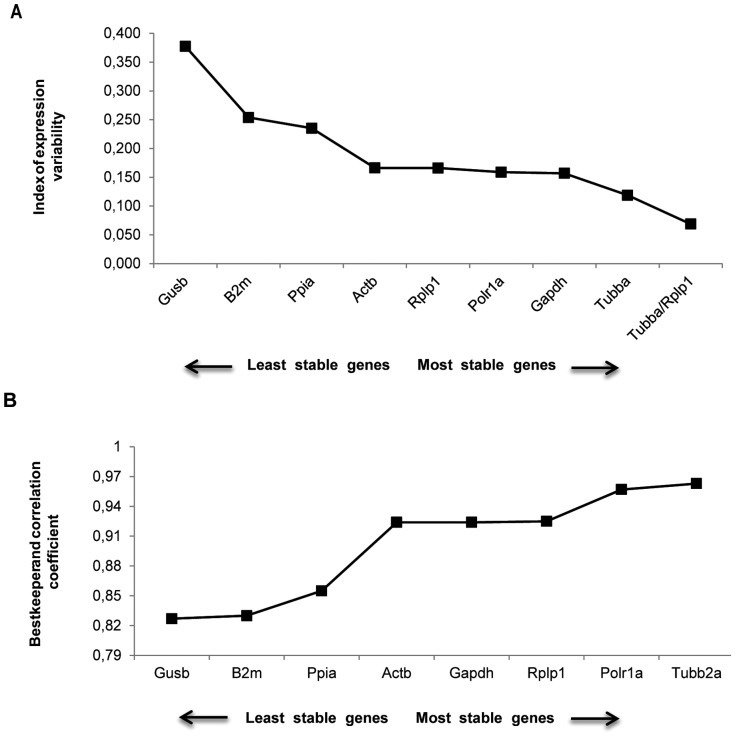
NormFinder and BestKeeper analysis of expression stability. Ranking of candidate reference genes based on stability values calculated by NormFinder (a) and BestKeeper (b) softwares for systemic PILO-model samples.

Results of NormFinder analysis are shown in Figure 3a. *Tubb2a, Rplp1, Gapdh, Polr1a* and *Actb* appeared as the most stable genes (stability between 0.144 and 0.174). *Gusb* was again the most unstable reference gene. The best combination of reference genes indicated was *Rplp1*/*Tubb2a*. These data sets are comparable with those obtained using geNorm, with slight differences in the ranking order of the most stable genes and of the best pair combination.

### Bestkeeper Analysis

Since all tested reference genes exhibit a standard deviation (SD) value lower than 1, none of them can be clearly considered inconsistent, and therefore all have been retained in the calculation of the BestKeeper índex (data not shown). The eight reference genes tested in our analysis correlated well one with another, and also when compared with the BestKeeper índex (Figure 3b). The best correlation between the reference gene and the BestKeeper index was obtained for *Tubb2a* (r = 0.938), followed by *Polr1a, Rplp1, Actb* and *Gapdh*. *Gusb* was again classified as the least reliable reference gene, exhibiting lower coefficient of correlation (r) than the Bestkeeper índex.

### Validation of reference genes

In order to validate results obtained for the reference genes, we conducted a relative expression analysis of the *Gfap* gene, whose mRNA expression pattern variation in the hippocampus of patient and animal models of MLT is known (21,32–39), comparing all experimental and control groups. We used each of the eight reference genes as internal controls as well as the recommended combination of genes from both geNorm and NormFinder.

**Figure 4:**
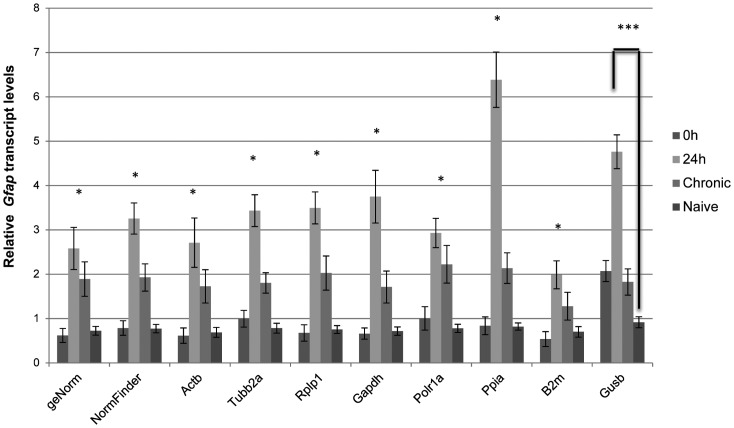
Relative quantities of Gfap in the hippocampus of systemic PILO injected rats upon different normalization approaches. qRT-PCR data were normalized by eight single reference genes and best combination derived by geNorm or NormFinder (mean ± SD), n = 6. The diagram shows mean levels of Gfap transcripts in epileptogenesis (0 h and 24 h), chronic period and animal naives. *24 h compared with 0h or naive group, p<0.05; ***24 h compared with naive group, p<0.05.

When normalized using individually *Actb, Rplp1, Gapdh, Ppia, Tubb2a, Polr1a* and *B2m* as reference genes, *Gfap* transcript was found to be significantly increased at 24 h compared with both 0 h and naive groups (Figure 4). Similar expression patterns were generated when either two of the most stable genes (as identified by geNorm or NormFinder) were used for normalization. Conversely, only when *Gusb* was employed for normalization, the difference in *Gfap* expression was not statistically significant between 0 h and 24 h groups. Curiously, normalization based on *Ppia* seems to accentuate the differences between 24 hours and the other groups.

### Evaluation of suitable reference genes in intrahippocampal PILO model

**Figure 5:**
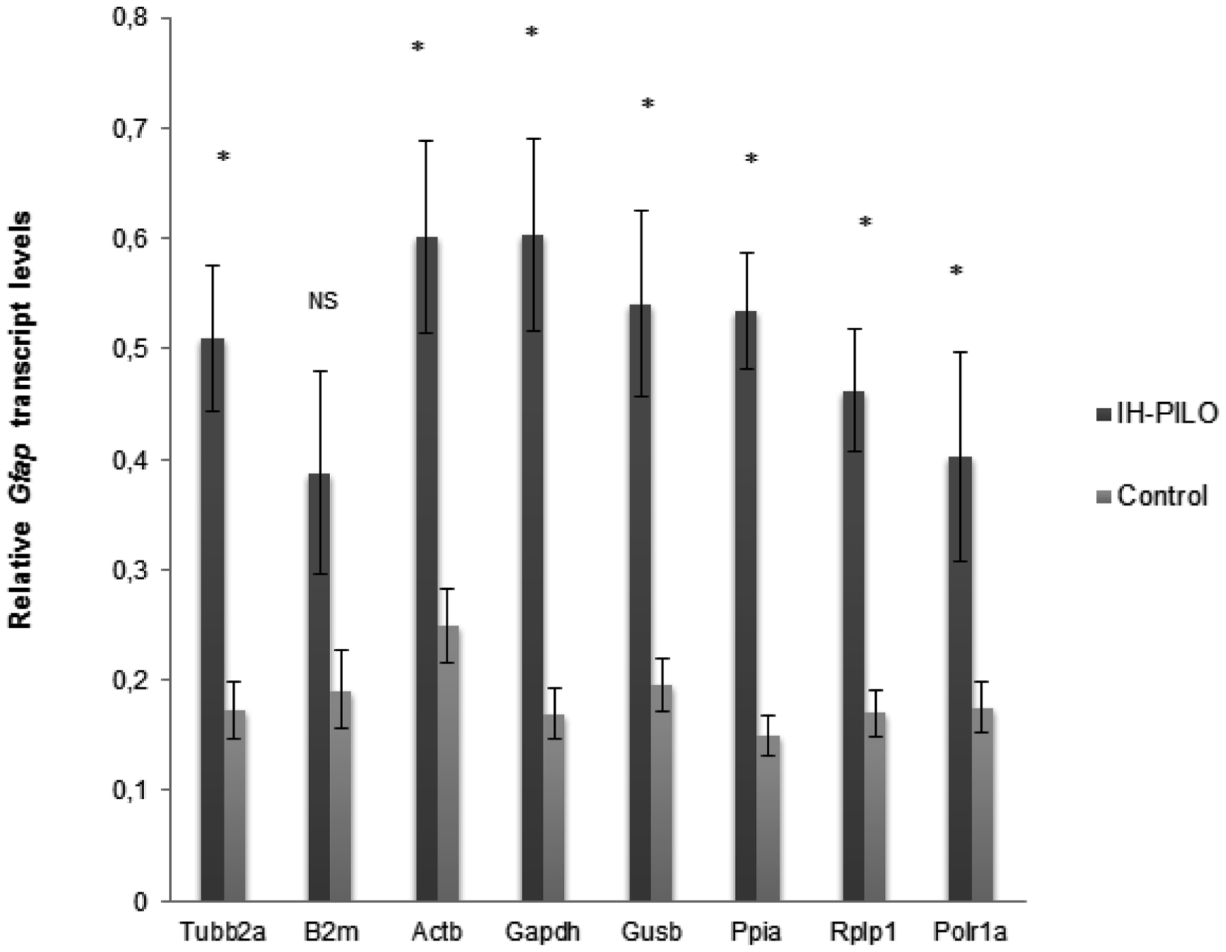
Relative quantities of Gfap in the hippocampus of intrahippocampal PILO injected rats using different reference genes for normalization. qRT-PCR data were normalized by eight single reference genes. The graphic shows mean levels (± SD) of Gfap transcripts in agude fase of epileptogenesis (experimental, n = 5) and controls (n = 6), * p<0.05.

In order to test the applicability of this candidate reference gene set on different experimental conditions, we performed a similar evaluation of suitable reference genes in acute phase of epileptogenesis induced by the injection of pilocarpine in the rat hippocampus. The comparison between the Ct raw data of intrahippocampal PILO injected and control groups showed significant differences for *B2m, Actb, Polr1a* and *Gusb*, but not for *Gapdh, Tubb2a, Ppia and Rplp1* (Figure S1). However, these differences are likely to be due to the preparation of the samples in the multistep process from tissue homogenization to RT-qPCR assay since analysis by geNorm, NormFinder and Bestkeeper indicated that all the analysed mRNAs were stable in the analysed conditions. Similarly to the systemic-PILO-model, all programs ranked *Act* and *Rplp1* as the most stable genes. In contrast, *B2m* was pointed out as the worst candidate gene by the three programs (data not shown). The relative expression of *Gfap*, normalized by selected reference genes, was compared between intrahippocampal PILO injected rats and controls (Figure 5). Interestingly, only when *B2m* was used as normalizer, the increase of *Gfap* mRNA was not revealed in the contralateral hippocampus of rats sacrificed 24 hours after SE.

## Discussion

It is well recognized that a reference gene should be properly validated for a particular experiment to ensure that gene expression is unaffected by the experimental condition. To the best of our knowledge, this is the first study that investigated suitable reference genes to different periods of the epileptogenic process in systemic and intrahippocampal pilocarpine induced models of MTLE. The possible differences between the pathways activated/suppressed in the induction of seizures in these two PILO-models are still uncertain. Systemic PILO is known to induce a cholinergic–glutamatergic coupling [40] which might also be true for the intrahipocampal PILO model. However, intrahippocampal-PILO-induced neurodegeneration is more selective than systemic PILO [41] indicating that there are different circuitries in thalamic, hypothalamic and limbic areas suffering from the hyperexcitability (or at least different cells susceptible to hyperexcitability) in these two PILO-models, which could have a differential impact on housekeeping gene expression.

The evaluation of a panel of eight candidate reference genes to determine the most reliable one for accurate normalization of gene expression in the systemic PILO-model indicates five (*Actb, Gapdh, Rplp1, Polr1a, Tubb2a*) as highly stable in the hippocampus of rats from all experimental and control groups. Depending on the software used (geNorm, NormFinder and Bestkeeper), the rank of these genes on a stability scale was slightly different, probably because of the different mathematical algorithm employed [24], [25]. However, all programs indicated *Gusb* as the most variable gene in our experimental setup. These results were supported by comparative analysis of the raw Ct values, because the highly ranked reference genes have narrower range of variations in expression levels among the experimental groups. Unlike, *Gusb*, the most unstable gene in the systemic PILO model, has a significant difference of expression between 24 and 0 h groups (Figure 1).

In order to evaluate the functional significance of the results obtained for reference genes, we conducted a relative expression analysis of the *Gfap* gene, whose pattern is already described for the PILO injected model. *Gfap*, an astrocyte-specific cytoskeleton protein, is used as a marker of reactive astrogliosis during epilepsy [42], [43], and is dramatically up-regulated in animal and human epileptogenic hippocampus [21], [32]–[39]. In fact, when normalized using *Actb, Rplp1, Gapdh, Ppia, Tubb2a, Polr1a, B2m* as reference genes, the *Gfap* transcript was found to be significantly increased at 24 h after PILO compared with other groups (Figure 4), which is consistent with the pattern of Gfap expression in different systems [21], [32]–[39]. Curiously, the use of *Ppia* as normalizer resulted in an overestimation of *Gfap* mRNA levels at this time window of epileptogenesis and the reason for this remains to be elucidated. Interestingly, under our experimental conditions, the use of *Gusb* for normalization leads the relative mRNA levels of the *Gfap* gene to be different from those obtained with stable genes, and hence probably less accurate. In a similar study based on the kainate-model, Pernot et al. [21] also observed that when *Gusb* was used as normalizer, the pattern of *Gfap* expression is lost. Our data corroborate, therefore, that *Gusb* is an unsuitable reference gene to normalizate relative expression data from various points of epileptogenesis. Our data also showed that *Gfap* mRNA levels did not change significantly at the chronic stage (Figure 4). While these data agree with studies in different experimental models [21], [36]–[38], other reports indicate that Gfap levels remain high during the chronic phase [32], [33], [39]. It is possible that these differences in the Gfap expression profile at chronic phase of espileptogenesis are related to individual variations in the levels of astrogliosis in the hippocampus. In fact, Garzillo & Melo [44] found that half of the animals subjected to pilocarpine-induced SE showed no reactive gliosis. We also considered whether selecting multiple reference genes in combination is better than selecting a single reference gene alone. The optimal number of reference genes which should be used for accurate normalization was determined by calculating the normalization factor (NF). The use of more than the two most stable reference genes identified (*Actb*/*Rplp1*) is not required as suggested by the V-value below the cut-off 0.15 which has been indicated by authors as the limit beneath which it would not be necessary to include additional reference genes [24] (Figure 3). In fact, normalization with only one of the stable genes seemed to provide comparable results for *Gfap* mRNA (time pattern and relative levels) to those obtained with the best combination of two genes pointed by NormFinder and geNorm programs. However, this cannot be inferred to be true for other mRNA or experimental conditions.

Following this rationale, and considering the consistency of the Systemic PILO data regarding the increase of the levels of *Gfap* at the acute phase of epileptogenesis, we evaluated the stability of the same candidate reference gene set in 24 hours after *SE* of epileptogenesis induced by intrahippocampal PILO injection. Curiously, all programs used (geNorm, NormFinder and Bestkeeper) included *Gusb* as a stable gene, whereas *B2m* was pointed out as the worst candidate gene. The results obtained to other reference genes were comparable to those observed for the systemic PILO model. These data were validated by analysis of *Gfap* relative expression in the hippocampus of rats. In fact, only when *B2m* was used as the normalizer, the increase of mRNA *Gfap* levels in 24 hours after SE was not detected. This emphasizes the concept that having a gene that is stable in one pathology model does not mean that the same gene will be stable in different experimental conditions related to this pathology. In fact, as observed in the intrahippocampal PILO model, when analysis is restricted to acute phase of epileptogenesis induced in the systemic PILO model, *B2m* (and not *Gusb*) is indicated as the most unstable gene by NormFinder, geNorm and Bestkeeper program, although this was not reflected in the quantification of *Gfap* expression (data not shown).

Differences in experimental design could explain the existence of some controversy among the most suitable and undesired genes to be used as references in MTLE gene expression studies. *Actb*, for instance, has shown to be a reliable reference gene in animal models of MTLE, but not in human epileptic brain tissue [20]–[23]. *Gapdh* was found to be a bad reference gene for expression analysis of both epileptogenesis induced by kainate and chronic phase in the PILO-model [21]–[23]. However, our study indicated *Gapdh* as a reliable reference gene as shown by stability expression results (using geNorm, NormFinder and Bestkeeper) and by validation based on *Gfap* expression analysis. Taking into account all these results, there is no single, universal, common optimal reference gene for expression analysis in MTLE and, therefore, the choice of reference genes should depend on the experimental condition under study.

## Conclusion

In summary, we investigated the suitability of eight potential candidate reference genes for normalization of gene expression during epileptogenesis induced in two different PILO-models of MTLE. We performed a stability expression analysis coupled to validation based on relative quantification of *Gfap* mRNA. Our results indicate that *Actb, Gapdh, Rplp1, Tubb2a* and *Polr1a* permit an efficient normalization for RT-qPCR studies across experimental condition under study. Moreover, *Gusb, B2m* and *Ppia* were unreliable normalizers in determined experimental conditions. Thus, it seems clear that the blind choice of reference genes without such evaluations should be avoided.

## Supporting Information

Figure S1 **Expression levels of the candidate reference genes in the hippocampus of intrahippocampal Pilo injected and control rats.** Values are given in the form of RT-qPCR threshold cycle numbers (Ct values), mean ± SD (experimental, n = 5 and control, n = 6), *24 h (Pilo-IH) compared with control group, p<0.05.
(DOCX)
