# Supplementary material for: Validation of Suitable Reference Genes for Expression Studies in Different Pilocarpine-Induced Models of Mesial Temporal Lobe Epilepsy
Source: PLoS One. 2013 Aug 23;8(8):e71892. doi: 10.1371/journal.pone.0071892 (PMC3751890; doi:10.1371/journal.pone.0071892)
Supplement: Figure S1 — Expression levels of the candidate reference genes in the hippocampus of intrahippocampal Pilo injected and control rats. Values are given in the form of RT-qPCR threshold cycle numbers (Ct values), mean ± SD (experimental, n = 5 and control, n = 6), *24 h (Pilo-IH) compared with control group, p<0.05. (DOCX) [file pone.0071892.s001.docx]

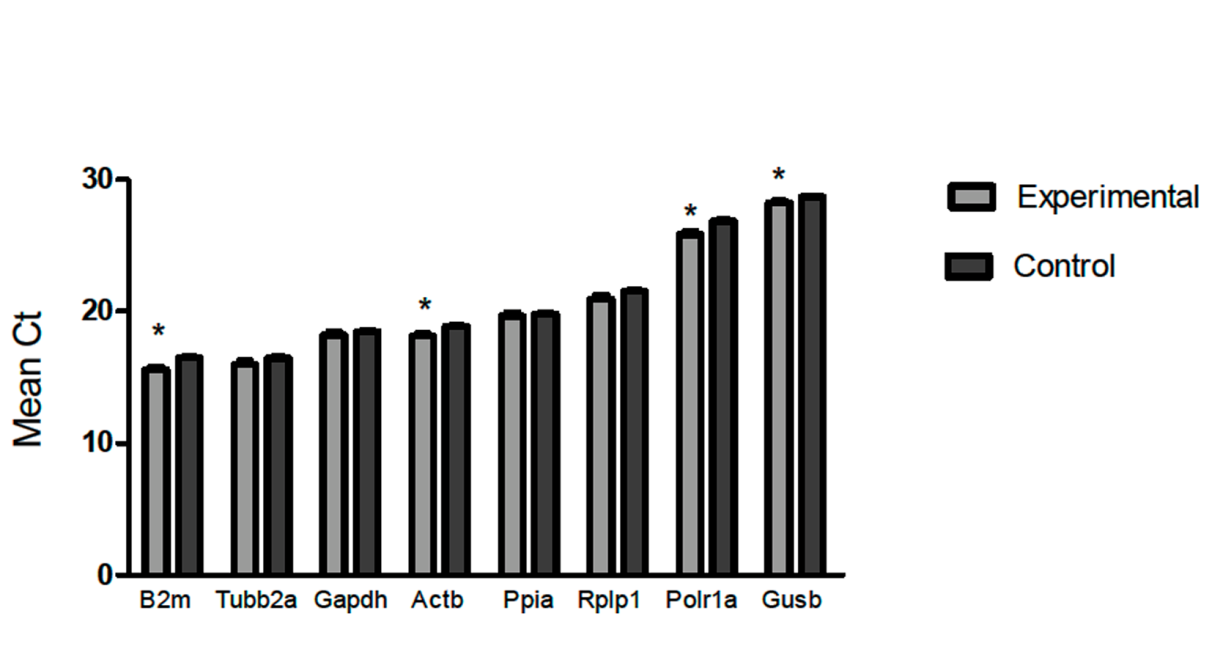


**Figure S1**. **Expression levels of the candidate reference genes in the hippocampus of intrahippocampal Pilo injected and control rats**. Values are given in the form of RT-qPCR threshold cycle numbers (Ct values), mean ± SD (experimental, n=5 and control, n=6), * 24h (Pilo-IH) compared with control group, p< 0.05.
